# A new unstable haemoglobin, Hb Alger, causes a transfusion‐dependent anaemia in early childhood

**DOI:** 10.1111/bjh.70040

**Published:** 2025-07-31

**Authors:** Syrine Bouazizi, Mathieu Cerino, Suzy Lim, Camille Desgrouas, Catherine Badens, Sarah Szepetowski, Nathalie Bonello‐Palot

**Affiliations:** ^1^ APHM, Molecular Genetics Laboratory, M2GM Biogenopole, Hôpital de la Timone Marseille Marseille France; ^2^ APHM, Biochemistry Department Biogenopole, Hôpital de la Timone Marseille France; ^3^ Aix Marseille Univ, INSERM, C2VN Marseille France; ^4^ AP‐HM, Pediatric Hematology Department Timone Children's Hospital Marseille France

**Keywords:** haemoglobin instability, haemoglobinopathy, *HBB* gene, paediatric anaemia

To the Editor,

Unstable haemoglobins are a group of pathological haemoglobin variants resulting from genetic variants affecting the genes encoding the globin chains (*HBA1* and *HBA2* for α chains and *HBB* for β chains). These variants, generally single nucleotide variations with autosomal dominant inheritance, alter the structure of haemoglobin, compromising its stability and biological function. These structural abnormalities lead to reduced solubility of the haemoglobin molecule and increased susceptibility to oxidation, resulting in the formation of intracellular protein precipitates known as Heinz bodies. These inclusions disrupt the integrity of the erythrocyte membrane, causing increased rigidity, fragility and premature destruction of red blood cells by the reticuloendothelial system, particularly in the spleen. In most cases, these processes result in chronic haemolytic anaemia, the severity of which varies depending on the degree of instability of the abnormal haemoglobin.^1^

Unstable haemoglobin can result from various genetic defects, such as missense variants, premature stop codons, deletions, insertions or frameshift variants, which can lead to elongated or truncated globin chains.^2^

In this study, we report the case of a 5‐year‐old Algerian patient referred for diagnosis and management of transfusion‐dependent anaemia. All protocols performed in this study complied with the ethics guidelines of the institutions involved.

The patient, from Algeria, was the third child of a non‐consanguineous couple. The mother had a history of three miscarriages. At birth, the patient's height and weight were within normal ranges. He developed neonatal jaundice requiring phototherapy. At 2 months of age, he presented with severe anaemia (haemoglobin 3 g/dL) and has required monthly transfusions since then. At 18 months, his total bilirubin was 102.6 μmol/L, haptoglobin was <0.8 μmol/L and Lactate Dehydrogenase was 5300 nkat/L. A bone marrow examination performed at 1 year showed erythroblastic hyperplasia and dyserythropoiesis, with no evidence of vitamin B9 or B12 deficiency. Due to severe splenomegaly, a splenectomy was performed at age of 4.

Upon his arrival in France at age of 5, the patient showed signs of significant iron overload, prompting the initiation of chelation therapy. He was treated with Exjade (450 mg/day) and Desferal (1400 mg, 5 days per week). The most recent ferritin level, measured in April 2025, was 673 pmol/L. Cardiac magnetic resonance imaging (MRI) revealed no abnormalities, while liver MRI showed significant iron accumulation.

Biochemical analysis of haemoglobin detected no anomalies, even when performed just prior to a transfusion, which was surprising because a diagnosis of thalassaemia was first suspected. Brilliant blue cresyl staining (methylene blue 1%) revealed Heinz bodies immediately, and after 1 and 2 h of incubation at 37°C (Figure 1). After informed consent was obtained from all individuals included in this study, molecular analysis identified a de novo heterozygous *HBB* c.193G>T (p.Gly65Cys) variant, which was not present in either parent. This variant is absent from the Genome Aggregation database and is predicted to be pathogenic by multiple bioinformatics tools. Whole Exome Sequencing revealed no additional anomalies. Structural modelling on the Mutation Explorer webserver (https://proteinformatics.uni‐leipzig.de/mutation_explorer/) estimated that the total energy of wild‐type haemoglobin was −377 and was increased to +4 for the mutant haemoglobin (p.Gly65Cys) (Figure 2).

**FIGURE 1:**
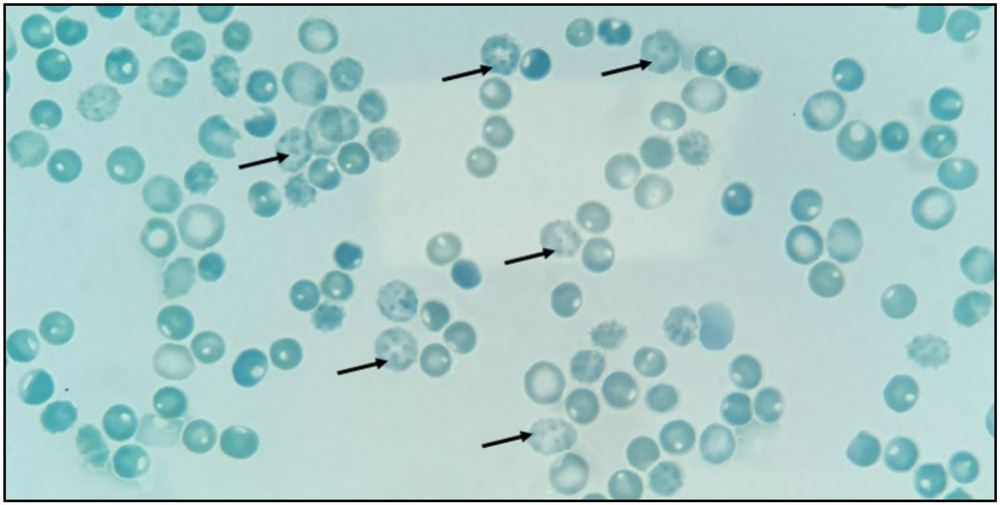
Visualisation of Heinz bodies in index case's blood film.

**FIGURE 2:**
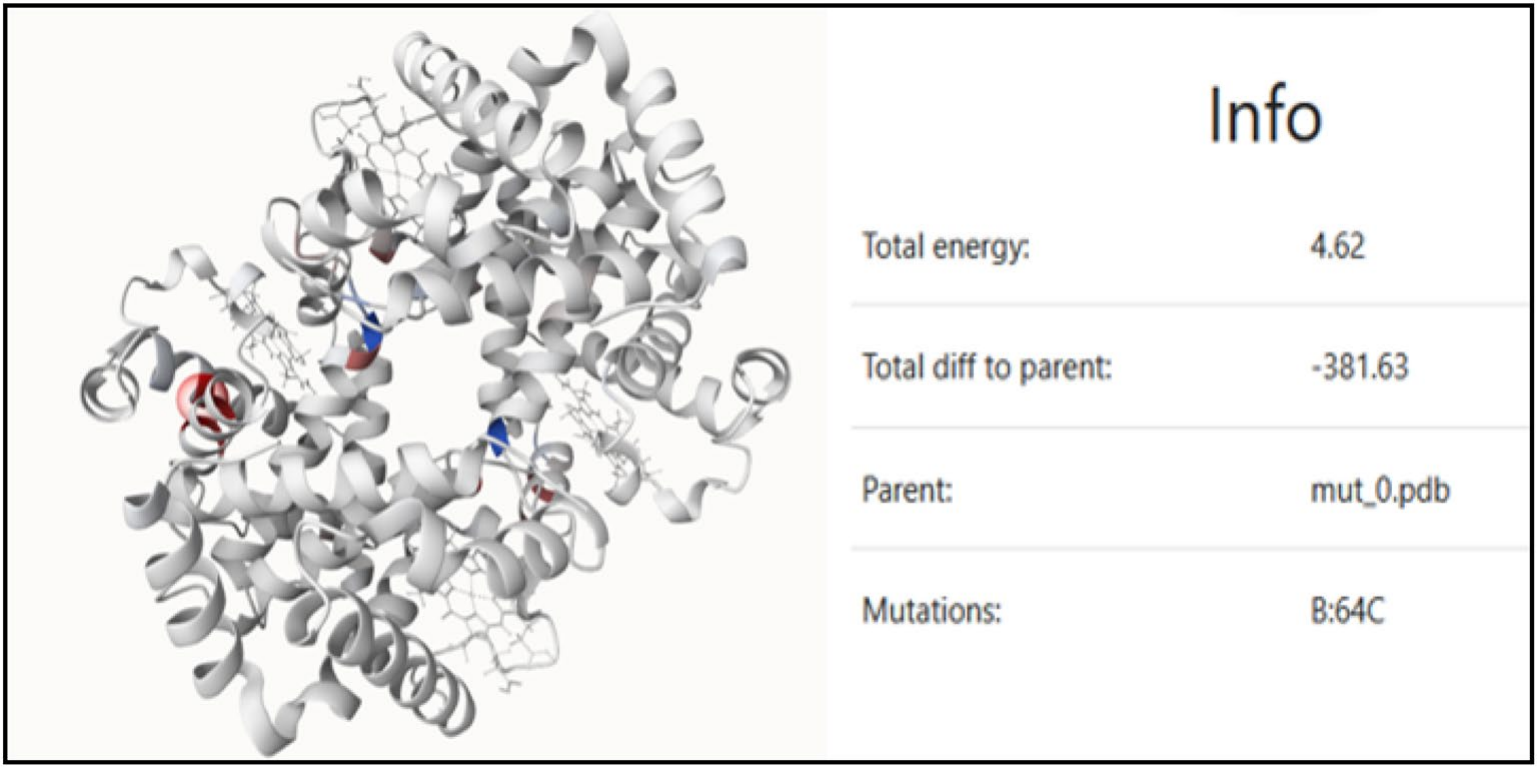
Mutation Explorer performed the mutation and calculated the energy of the structure (PDB: 7XGY) as the sum of energies computed for each residue. The red circle indicated the mutated residue and the difference colouring showed the mutant (mutated result structure) minus the wild type (WT) (parent structure) energy for each residue (with negative values in blue and positive values in red). It estimated that the total energy of wild‐type haemoglobin was −377 and was increased to +4 for the mutant haemoglobin (p.Gly65Cys).

The c.193G>T variant is in a region of the *HBB* gene known to be associated with haemoglobin instability. Notably, three other variants have been reported at codon 65: c.194G>A (p.Gly65Asp),^3^ c.194G>C (p.Gly65Ala)^4^ and c.194G>T (p.Gly65Val).^5^ These variants all affect the glycine residue at position 65 a highly conserved amino acid that plays a crucial role in maintaining the structural integrity of haemoglobin. A point is that de novo mutations occur most commonly at CpG dinucleotides, higher by a factor of ~18% than other dinucleotides, particularly at glycine and arginine, which account for ~40% of these nonsynonymous mutations, due to spontaneous deamination of methylated cytosine to thymine.^6^ In our case, substitution of this glycine alters the protein's conformation and induces instability, potentially leading to various clinical outcomes depending on the nature of the amino acid substitution.^1^ This suggests a correlation between the biochemical properties of the substituted residue and the severity of the disease.^7^

The p.Gly65Asp and p.Gly65Ala variants are generally associated with moderate anaemia, rarely requiring transfusions.

The p.Gly65Asp variant, known as haemoglobin J‐Calabria, is found in individuals with haemoglobin levels around 10 g/dL and minimal need for transfusions. The substitution introduces a negatively charged carboxyl group, which may interfere with electrostatic interactions in the haemoglobin molecule. However, the slight size difference between aspartate and glycine likely limits the overall destabilising effect.^3^

The p.Gly65Ala variant, known as haemoglobin Aubagne, has been observed in a case of persistent anaemia during a second pregnancy, with haemoglobin levels around 8.5 g/dL. Transfusion needs are also minimal. In this case, glycine is replaced by alanine, a small yet hydrophobic residue. While less disruptive than aspartate, alanine may still affect protein packing and reduce haemoglobin stability.^4^

In contrast, the p.Gly65Val variant, known as haemoglobin Calgary, leads to severe haemolytic anaemia and dyserythropoiesis, with haemoglobin levels around 7.6 g/dL and monthly transfusion requirements. The replacement of glycine with valine, a larger, more hydrophobic amino acid, significantly disrupts the hydrophobic core and spatial structure of the haemoglobin molecule, contributing to its instability and the severity of clinical symptoms.^7^

In our case, glycine is replaced by cysteine, a larger, polar amino acid featuring a reactive thiol (–SH) group capable of forming disulphide bonds. This chemical property can dramatically alter haemoglobin's three‐dimensional structure, potentially causing greater instability than previously reported substitutions. Depending on its location and context within the protein, cysteine may interfere with proper tetramer formation or promote aberrant disulphide linkages. These disruptions can increase the molecule's susceptibility to degradation or aggregation, thereby exacerbating haemolysis and anaemia.^8^

Computational modelling revealed increased energy levels for all four mutated haemoglobins compared to the wild‐type form, the most symptomatic variants exhibiting the highest differences with the wild‐type haemoglobin. Since lower energy values indicate greater structural stability, this significant energy increase suggests a substantial decrease in haemoglobin stability (Table 1).

**TABLE 1:** The different reported variants in codon 65 linked to unstable haemoglobin.

Hb variants	Hb name	Clinical symptoms	Hb level (g/dL)	Transfusion regimen	Total energy of haemoglobin	References
*c.194G>A* (*p.Gly65Asp*)	Hb J‐calabria	Completely asymptomatic	Hb ≃ 10	Rare	−25	3
*c.194G>T* (*p.Gly65Val*)	Hb Calgary	Severe haemolytic anaemia and dyserythropoiesis	Hb = 7.6	Monthly	+398	5
*c.194G>C* (*p.Gly65Ala*)	Hb Aubagne	Persistent anaemia during pregnancy	Hb = 8.5	Rare	−297	4
*c.193G>T* (*p.Gly65Cys*)	Hb Alger	Severe anaemia since the age of 2 months, splenomegaly	Hb = 6	Monthly	+4	Our study

Abbreviation: Hb, haemoglobin.

We have identified and characterised a novel variant in the *HBB* gene, named Haemoglobin Alger, which results in severe anaemia with both haemolytic and dyserythropoietic features, necessitating a regular transfusion regimen in the first year of life.

In this particular case, molecular analysis has thus proved to be a crucial step in both diagnosis and therapeutic management since it was instrumental not only in clarifying the aetiology of the anaemia but also in enabling the consideration of a curative treatment by haematopoietic stem cell transplantation, the patient's father being a compatible donor.

## AUTHOR CONTRIBUTIONS

SB performed the research. SS and CB designed the research study. MC and SL contributed essential reagents or tools. CD estimated the total energy of the different haemoglobins. NB‐P analysed the data; SS and NB‐P wrote the paper.

## FUNDING INFORMATION

This research work was not supported.

## CONFLICT OF INTEREST STATEMENT

The authors report no competing interests.

## References

[1] Maeda M , Yamamoto M . The unstable hemoglobin disease. Nihon Rinsho. 1996;54(9):2436–2441. 8890575

[2] Préhu C , Pissard S . Two French Caucasian families with dominant thalassemia‐like phenotypes due to hyper unstable hemoglobin variants: Hb Sainte Seve [codon 118 (‐T)] and codon 127 [CAG‐‐>TAG (Gln‐‐>stop)]. Hemoglobin. 2005;29(3):229–233. 10.1081/hem-200066335 16114188

[3] Marinucci M , Mavilio F , Fontanarosa PP , Tentori L , Brancati C . Studies on a family with Hb J Calabria (alpha 2 beta 2 64 (E8) Gly replaced by Asp). Hemoglobin. 1979;3(5):327–340. 500375 10.3109/03630267908997538

[4] Lacan P , Badens C . Hb aubagne [beta64(E8)Gly‐Ala]: a new unstable beta chain variant found in a French family. Hemoglobin. 2002;26(2):163–167. 10.1081/hem-120005454 12144059

[5] Martin G , Grimholt RM . Hb calgary: a highly unstable hemoglobin variant with a β‐thalassemia major phenotype. Hemoglobin. 2021;45(4):215–219. 34311670 10.1080/03630269.2021.1956947

[6] Ohno M . Spontaneous de novo germline mutations in humans and mice: rates, spectra, causes and consequences. Genes Genet Syst. 2019;94(1):13–22. 10.1266/ggs.18-00015 30381610

[7] Huisman TH , Brown AK , Efremov GD , Wilson JB , Reynolds CA , Uy R , et al. Hemoglobin Savannah (B6(24) beta‐glycine is greater than valine): an unstable variant causing anemia with inclusion bodies. J Clin Invest. 1971;50(3):650–659. 10.1172/JCI106535 5545125 PMC291973

[8] Thom CS , Dickson CF . Hemoglobin variants: biochemical properties and clinical correlates. Cold Spring Harb Perspect Med. 2013;3(3):a011858. 10.1101/cshperspect.a011858 23388674 PMC3579210

